# African Swine Fever Virus Ubiquitin-Conjugating Enzyme Is an Immunomodulator Targeting NF-κB Activation

**DOI:** 10.3390/v13061160

**Published:** 2021-06-17

**Authors:** Lucía Barrado-Gil, Ana del Puerto, Inmaculada Galindo, Miguel Ángel Cuesta-Geijo, Isabel García-Dorival, Carlos Maluquer de Motes, Covadonga Alonso

**Affiliations:** 1 Department of Biotechnology, Instituto Nacional de Investigación y Tecnología Agraria y Alimentaria (INIA-CSIC), Ctra. de la Coruña km 7.5, 28040 Madrid, Spain; barrado.lucia@inia.es (L.B.-G.); delpuerto.ana@inia.es (A.d.P.); galindo@inia.es (I.G.); cuesta.miguelangel@inia.es (M.Á.C.-G.); isabel.garcia@inia.es (I.G.-D.); 2 Department of Microbial Sciences, School of Biosciences and Medicine, University of Surrey, Stag Hill, Guildford GU2 7XH, UK

**Keywords:** innate immunity, TLR, NF-κB, AP-1, IFN, STING, African swine fever virus, ubiquitin, E2 enzyme, ubiquitin-conjugating enzyme, ASFV

## Abstract

African swine fever virus (ASFV) is an acute and persistent swine virus with a high economic burden that encodes multiple genes to evade host immune response. In this work, we have revealed that early viral protein UBCv1, the only known conjugating enzyme encoded by a virus, modulates innate immune and inflammatory signaling. Transient overexpression of UBCv1 impaired activation of NF-κB and AP-1 transcription factors induced by several agonists of these pathways. In contrast, activation of IRF3 and ISRE signaling upon stimulation with TRIFΔRIP, cGAS/STING or RIG-I-CARD remained unaltered. Experiments aimed at mapping UBCv1 inhibitory activity indicated that this viral protein acts upstream or at the level step of IKKβ. In agreement with this, UBCv1 was able to block p65 nuclear translocation upon cytokine stimulation, a key event in NF-ĸB signaling. Additionally, A549 stably transduced for UBCv1 showed a significant decrease in the levels of NF-ĸB dependent genes. Interestingly, despite the well-defined capacity of UBCv1 to conjugate ubiquitin chains, a mutant disabled for ubiquitylation activity retained similar immunomodulatory activity as the wild-type enzyme, suggesting that the two functions are segregated. Altogether these data suggest that ASFV UBCv1 manipulates the innate immune response targeting the NF-κB and AP-1 pathways and opens new questions about the multifunctionality of this enzyme.

## 1. Introduction

The initiation of the innate immune response is based on the recognition of invading viruses by host cells via the detection of pathogen-associated molecular patterns (PAMPs). To this end, a cooperative array of host-encoded pattern recognition receptors (PRRs) activates cellular signal transduction pathways to coordinate an effective response against viruses [1,2]. The transcription factor nuclear factor kappa light-chain-enhancer of activated B cells (NF-κB) as well as interferon (IFN) regulatory factors (IRF) are key transcription factors in innate immune activation that regulate the expression of cytokines and chemokines, including type I IFN (IFNα, IFNβ), and coordinate an effective antiviral immune response. NF-κB is activated upon engagement of PRRs such as Toll-like receptors (TLRs) or the receptors of inflammatory cytokines such as tumor necrosis factor-α (TNF-α) or interleukin-1β (IL-1β). The activated signaling pathways downstream of these receptors involve common intermediates including the adaptor proteins TNF receptor associated factor (TRAF) and the kinase complex phosphorylating the inhibitor of ĸB (IκB), with its 3 subunits IKKα, IKKβ and IKKγ [3,4]. Ubiquitylation is a relevant process in the regulation of signaling cascades of the innate immune response, particularly in the control of NF-ĸB activation [5,6]. In this pathway the formation of Lys 63-linked ubiquitin (Ub) chains activates the transforming growth factor-β activated kinase 1 (TAK1) complex and similarly, the formation of Met 1-linked chains mediates activation of the IKK complex [3,7]. Activated IKK phosphorylates IκBα, predominantly via the action of the catalytic subunit IKKβ, an event that triggers its Lys 48-linked polyubiquitination and proteasomal degradation. In resting cells, IκB retains NF-κB complex subunits p65 and p50 in their inactive form in the cell cytosol. Thus, its proteasomal degradation results in NF-ĸB nuclear translocation and the initiation of NF-ĸB-dependent gene expression [8,9].

In addition to NF-ĸB, the host cell antiviral response also includes the activation of IRFs and mitogen-activated protein kinases (MAPK) signaling. Many TLRs induce the activation of IRF3, including IRF3, which can be activated downstream of the adaptor TIR domain-containing adaptor-inducing IFN-β (TRIF) and TLR3. Furthermore, IRF3 can be activated by cytosolic sensors, such as the RNA sensor retinoic acid–inducible gene I (RIG-I) or the DNA sensor cyclic GMP-AMP synthase (cGAS) [10,11]. These sensors associate with the intracellular adaptors mitochondrial antiviral signaling protein (MAVS) and stimulator of IFN genes (STING), respectively, to drive IRF3 activation. Activating protein-1 (AP-1) transcription factor also contributes to optimal type I IFN production and can be hijacked by viruses [12]. A critical protein in the activation of the mitogen-activated protein kinase (MAPK) pathway and AP-1 is the kinase TAK1, which acts as a convergent point for both AP-1 and NF-κB signaling [13]. The mammalian MAPK pathway results in the activation of extracellular signal-regulated kinases (ERKs), c-Jun N-terminal kinases (JNKs) and p38 MAPKs, which collectively regulate numerous proteins and/or transcription factors related to immune response [14].

Given the importance of these pathways in innate immune activation, viruses have evolved a wide range of countermeasures that avoid or tolerate these cellular responses. This is particularly evident in large DNA viruses, which devote large proportions of their coding capacity to targeting the host immune system to promote infection. ASFV produces a deadly infection of swine that is spreading in three continents (Asia, Europe and Africa) with high economic burden. At present, there is not a commercial vaccine available, although live attenuated vaccines are being developed by deletion of genes related to the viral manipulation of the immune system [15,16]. Hence, a better understanding of how ASFV evades host immune responses has the potential to impact in the rational design of these vaccines. ASFV has developed several mechanisms to influence the innate immunity. ASFV encodes an IκB homolog, the A238L protein, that inhibits transcriptional activation of NF-κB [17,18] and other transcription factors (NFAT, cJun, cFos) thereby inhibiting the expression of several proinflammatory cytokines [19]. A238L also inhibits p65/RelA acetylation, and the transcriptional coactivators of p65, p300 and CREB-Binding protein, thereby inhibiting the expression of inflammatory regulators cyclooxygenase-2 (COX-2) and TNFα [20,21,22]. Another example is the A276R protein from multigene family (MGF) 360 that inhibits the induction of interferon-β (IFN-β) via TLR3 and cytosolic pathways, affecting IRF3 or NF-κB [23]. Another TLR3 homolog is the I329L protein, inhibiting IFN induction at the level of TIR-domain-containing adapter-inducing interferon-β (TRIF) [24]. Finally, A258R, a member of the MGF530 family, inhibits both IFNβ and NF-ĸB pathways of the type I IFN stimulation [23,25]; and the L86L open reading frame (ORF) encodes for a non-essential gene and has been described to interact with host protein IL-1β, although the implications in innate immune response are unknown [26].

The ASFV *I215L* gene codes for the only known ubiquitin-conjugating enzyme encoded by a virus. It is expressed very early during infection and it is able to associate with several classes of polyubiquitin chains. This activity is dependent on its catalytic domain [27]. In addition, UBCv1 function is essential for infection given that the knockdown of this gene by siRNA impaired viral infection [28]. In this paper, we studied the activity of the viral ubiquitin conjugase UBCv1 in innate immune activation. The results presented here demonstrate that transient transfection of UBCv1 (and a conjugating-inactive mutant) impairs the ability of host cells to activate NF-κB and AP-1, but not IRF-3, signaling pathways and that this activity maps upstream of the IKK complex. These data indicate that UBCv1 is able to dampen inflammatory signaling in isolation irrespective of its ubiquitylating function and poses future questions about the multifunctionality of this protein.

## 2. Materials and Methods

### 2.1. Cell Culture

A549 cells (ATCC^®^ CCL-185, Manassas, VA, USA) and HEK293T cells (ATCC^®^ CRL-11268, Manassas, VA, USA) were grown at 37 °C in a 5% CO_2_ atmosphere in Dulbecco’s Modified Eagle’s Medium (DMEM, Gibco, Waltham, MA, USA) supplemented with 10% fetal bovine serum (FBS, Gibco, Waltham, MA, USA), 100 IU/mL penicillin, 100 μg/mL streptomycin and GlutaMAX (Gibco, Waltham, MA, USA). A549-empty and A549-UBCv1 stable cell lines were grown as above with the addition of 5 µg/mL puromycin (Gibco, Waltham, MA, USA). Vero cells (ATCC^®^ CCL-81, Manassas, VA, USA) were grown with 5% FBS.

### 2.2. Expression Vectors and Reagents

Flag-tagged UBCv1 and UBCv1mutated in C85A (UBCv1mut) were cloned into a pcDNA4/TO vector (Invitrogen, Carlsbad, CA, USA) as previously described [27]. All plasmids for signaling molecules and reporter genes including VACV proteins C6 and A49 have been previously described [29,30,31,32].

Transfections were achieved using polyethylenimine (PEI; Sigma-Aldrich, Saint Louis, MO, USA) as described below or Lipofectamine 2000 (LF2000; Invitrogen, Carlsbad, CA, USA) following manufacturer’s instructions.

HEK293T, A549-empty cells and A549-UBCv1 cells were treated with 25–50 ng/mL hIL-1β (Peprotech, Rocky Hill, NJ, USA), 10 ng/mL PMA (phorbol 12-Myristate 13-Acetate, Santa Cruz Biotechnology- SCBT, Dallas, TX, USA) for 8 h, 25–50 ng/mL hTNFα for 6–8 h, or 500 U/mL IFNα for 8 h (both from Peprotech).

### 2.3. Overexpression of UBCv1 in Cell Lines

To generate A549 cells overexpressing, UBCv1 was amplified by PCR using primers 5′ GACGGATCCATGGATTACAA -3′ (Fw) and 5′- GCGCTCTAGA TTACTCATCATC -3′ (Rv). The PCR product was subcloned into the commercially lentiviral expression vector pLVX-Puro (Clontech, Mountain View, CA, USA). Lentiviral particles were produced by transfection of HEK293T cells (seeded in 60 mm dishes) with 5 μg of pLVX, 3 μg of psPAX2 packaging plasmid (Addgene 12260, Watertown, MA, USA), and 3 μg of vesicular stomatitis virus-G glycoprotein using Lipofectamine 2000 (Life Technologies, Carlsbad, CA, USA). Supernatants containing the lentivirus were harvested at 48 and 72 h post transfection, 0.45 μM-filtered, and used to transduce A549 cells, which were subsequently selected for puromycin resistance (Life Technologies, 5 μg/mL).

### 2.4. Western Blot Analysis

Cells were harvested in modified RIPA buffer (50 mM TrisHCl pH7.4, 1 mM EDTA, 1 mM EGTA, 100 mM NaCl, 1% Triton X100, 0.2% sodium deoxycholate and 0.1% SDS) supplemented with 1× proteases inhibitors (cOmplete Mini, EDTA free protease inhibitor cocktail tablets, Roche, Basel, Switzerland) and 1× phosphatases inhibitors (PhosSTOP EASY pack, Roche). The lysates were incubated 20 min on ice and then spun at 15,000× *g* for 10 min at 4 °C to eliminate cellular debris.

Once the proteins were electrophoretically separated, they were transferred to a 0.45 µm nitrocellulose membrane (Amersham Biosciences, Piscataway NJ, USA) at 100 V during 90 min. Blocking was made in 5% skimmed milk powder dissolved in TBS-0.1% Tween (TBS-T) (50 mM Tris-HCl (pH 8.3), 150 mM NaCl and 0.5% (*v/v*) Tween-20) buffer for 1 h at room temperature.

We used the following primary antibodies: mouse monoclonal anti-tubulin (Sigma-Aldrich, Saint Louis, MO, USA) and rabbit polyclonal antibody against UBCv1 [27]. The primary antibodies were diluted in 5% skimmed milk powder dissolved in TBST at desired concentration and then incubated at 4 °C overnight. After three washes, blots were incubated with appropriate HRP secondary antibody diluted in 2% skimmed milk powder dissolved in TBST at 1:5000 for 1 h at room temperature. Blots then were developed using enhanced chemiluminescence reagent (Bio-Rad, Hercules, CA, USA) and detected with ChemiDoc™ XRS Gel Imaging System using Image Lab™ software (Bio-Rad).

### 2.5. Immunofluorescence

Cells were seeded onto 13 mm glass coverslips in 24 well plates prior to transfection and agonist treatment. Then, cells were washed with PBS and fixed with 4% paraformaldehyde (PFA) for 15 min. After a PBS wash, cells were permeabilized 10 min with 0, 1% Triton X-100 in PBS. Then, coverslips were washed with PBS and incubated for 1 h in 2% bovine serum albumin (BSA, Sigma-Aldrich, Saint Louis, MO, USA) diluted in PBS. Slides were then incubated for 1 h in primary antibody diluted in 1% BSA in PBS. We used the following primary antibodies: mouse monoclonal anti-p65 (SCBT, Dallas, TX, USA) and rabbit anti-Flag (Sigma-Aldrich, Saint Louis, MO, USA) The appropriate secondary antibody conjugated to Alexa Fluor −488 or −594 (ThermoFisher, Waltham, MA, USA) was used and cell nuclei was detected with TOPRO3 (ThermoFisher). Coverslips were mounted on glass slides using ProLong Gold (ThermoFisher). Cells were visualized using TCS SPE confocal microscope (Leica, Wetzlar, Germany) with a 63× Oil immersion objective. The Image acquisition was performed with a Leica Application Suite Advanced Fluorescence software (LAS AF). All the images were taken with a 1024 × 1024 pixels’ resolution. For immunofluorescence quantitative analysis of p65 translocation, fluorescence intensity of specific antibody was measured using ImageJ 1.53c software (NIH). Specific ROIs were defined for 20 transfected.

### 2.6. Reporter Gene Assays

HEK293T cells were seeded in 96-well plates and transfected with reporter plasmids containing the firefly luciferase gene (driven by the promoter of interest) and the constitutively active Renilla luciferase plasmid. The firefly luciferase reporters and the quantity used in the current study are indicated in the results section. For the transfection protocol, PEI was mixed with Opti-MEM (Life Technologies, Carlsbad, CA, USA) following the manufacturer’s protocol. Each pathway was stimulated by co-transfection with activating plasmids or with different agonists after 24 h of transfection. In both cases, cells were then lysed with 100 μL of 1× Passive Lysis Buffer (PLB; Promega, Madison, WI, USA). Firelfy luciferase (FLuc) and Renilla luciferase (RLuc) activity was measured in a Clariostar plate reader (BMG Biotech, Ortenberg, Germany) using manufacturer’s protocol. FLuc/RLuc ratios were calculated for each well and normalized to empty vector (EV) samples as a fold increase. Experiments were performed in quadruplicate and repeated at least three times. Plasmids for signaling molecules and reporter genes are described in results.

### 2.7. Quantitative PCR

RNA was extracted from A549 cells grown in 6-well plates using RNeasy RNA extraction kit (QIAgen, Hilden, Germany) according to the manufacturer’s protocol. For retrotranscription QuantiTect Reverse Transcription kit (QIAgen) was used to synthesize cDNA, also following the manufacturer’s protocol. 250 ng of cDNA were used as the template for real-time PCR using QUANTITECT SYBR GREEN PCR KIT (QIAgen). Reactions were performed using the ABI 7500 Fast Real-Time PCR System (Applied Biosystems, Waltham, MA, USA). Expression of each gene was normalized to an internal control (*GAPDH* [glyceraldehyde-3-phosphate dehydrogenase]), and these values were then normalized to the value for the nonstimulated mock-infected control cells to yield the fold induction. The following primers were used: hGAPDH_fwd (5′ ACC CAG AAG ACT GTG GAT GG 3′), hGAPDH_rev (5′ TTC TAG ACG GCA GGT CAG GT 3′), hICAM-1_fwd (5′ TCT GTG TCC CCC TCA AAA GTC 3′), hICAM-1_rev (5′ GGG GTC TCT ATG CCC AAC AA 3′), hCCL2_fwd (5′ CAG CCA GAT GCA ATC AAT GCC 3′), hCCL2_rev (5′ TGG AAT CCT GAA CCC ACT TCT 3′), UBCv1_fwd (5′ GAT GCAGCTAAAAGCTACCGT 3′), and UBCv1_rev (5′ TGGTGGAACATTGGATGCAG 3′).

### 2.8. Statistical Analysis

The experimental data were analysed by one-way ANOVA by GraphPad Prism 8 (GraphPad Software, San Diego, CA, USA). For multiple comparisons, Bonferroni´s post-test correction was applied. Values were expressed in graph bars as mean ± SD of at least three independent experiments unless otherwise noted. A *p* value < 0.05 was considered statistically significant.

## 3. Results

### 3.1. UBCv1 Inhibits NF-κB Activation

**Figure 1:**
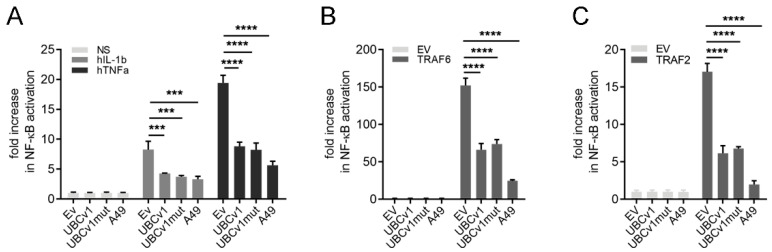
UBCv1 inhibited NF-κB activation. (**A**) HEK293T cells were seeded in 96-well plates and transfected in quadruplicates with firefly luciferase reporter plasmid under the control of a NF-κB-dependent promoter (70 ng/well), p-TK-Renilla luciferase transfection control (10 ng/well), and 25 ng/well of UBCv1, UBCv1mut, positive control A49 expression plasmids or empty vector control (EV). 24 h after transfection cells were treated with 50 ng/mL TNFα or 25 ng/mL IL-1β for a further 8 h (**B**,**C**) HEK293T were transfected as above, but including 10 ng/well of TRAF2 or TRAF6. Firefly luciferase activity was measured 24 h post- transfection and corrected with Renilla luciferase activity and normalized to EV. Data are presented as mean ± SD and are representative of at least three experiments. Statistical analyses were performed using one-way ANOVA test (*** *p* < 0.001; **** *p* < 0.0001).

We set out to determine the ability of the viral enzyme UBCv1 to modulate immune responses. Initially, we assayed NF-κB activation in response to stimuli such as hIL-1β or hTNFα. HEK 293T cells were transfected with an NF-κB-luciferase reporter alongside with a 3XFlag-tagged UBCv1 or UBCv1 mutated in the cystein residue responsible of the catalytic activity (UBCv1mut) [27]. These cells were subsequently stimulated with 25 ng/µL IL-1β or 50 ng/µL TNFα for 6 h (Figure 1). As a positive control we used vaccinia virus (VACV) protein A49, a protein known to prevent p65 nuclear translocation by mimicking IĸBα and blocking its proteasomal degradation [30,33,34]. Expression of UBCv1 decreased NF-κB activation in a statistically significant manner compared to cells transfected with an empty vector (EV) control (Figure 1A). This inhibition was also detected upon expression of VACV protein A49.

NF-κB activation was also stimulated by over-expression of the pathway adaptor proteins TRAF6 and TRAF2. Under these conditions UBCv1 yielded similar results and inhibited reporter activity by >50% compared to EV-transfected cells (Figure 1B,C). As expected, this inhibition was also observed for VACV A49. Interestingly, the catalytically inactive mutant C85A (referred to as UBCv1mut) retained the same inhibitory potential as the wild-type protein. Taken together, these results revealed that UBCv1 modulates transcription factor NF-κB activation and this action was not dependent on UBCv1 C85A residue.

### 3.2. UBCv1 and UBCv1mut Do Not Affect Type I IFN Signaling

**Figure 2:**
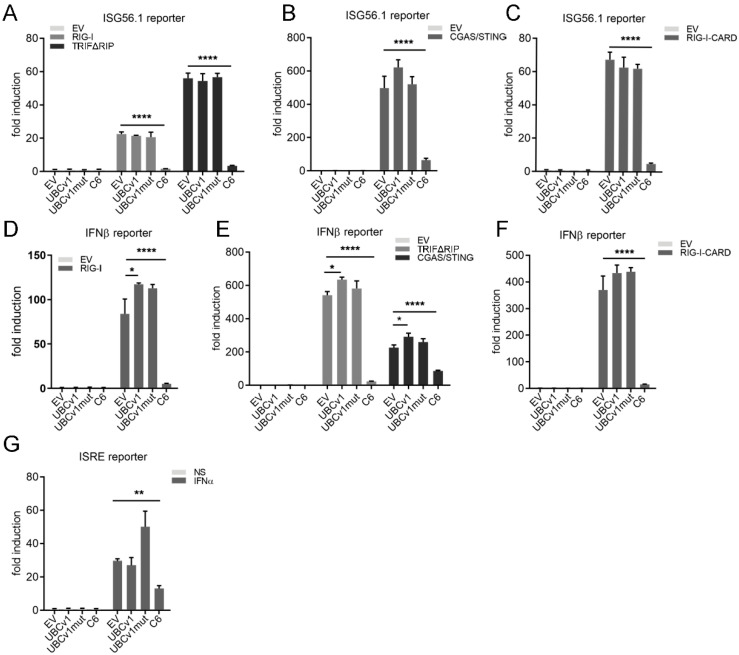
UBCv1 did not affect IRF3 and ISRE signaling. HEK293T cells seeded and transfected with a reporter plasmid expressing firefly luciferase under the control of the ISG56 promoter (70 ng/well), p-TK-Renilla (10 ng/well) and either UBCv1, UBCv1mut, C6 or empty vector (EV) at 25 ng/well. To stimulate the pathway, cells were co-transfected with the following expression vectors for (**A**) RIG-I (40 ng/well), TRIFΔRIP (40 ng/well), (**B**) cGAS (20 ng/well) and STING (20 ng/well) or (**C**) RIG-I-CARD (5 ng/well). (**D**) HEK293T cells transfected as above but using a reporter plasmid expressing firefly luciferase under the control of the IFNβ promoter and the following inducers (**D**) RIG-I, (**E**) TRIFΔRIP, cGAS and STING or (**F**) RIG-I-CARD. (**G**) HEK293T cells were transfected with pISRE-luc reporter (70 ng/well), p-TK-Renilla luciferase (10 ng/well) and expression vectors for UBCv1, UBCv1mut and C6 proteins (25 ng/well). After 24 h cells were stimulated with 500 U/mL IFNα for a further 8 h. Firefly luciferase activity was measured 24 h post transfection and corrected with Renilla luciferase activity and normalized to EV. Data are presented as mean ± SD and are representative of at least three experiments. Statistical analyses were performed using one-way ANOVA test (* *p* < 0.05; ** *p* < 0.01; **** *p* < 0.0001).

In order to determine the specificity of action on the innate immune response we transfected HEK293T cells with a reporter expressing luciferase under the promoter of the IRF3-dependent gene *ISG56*. The pathway was stimulated by co-transfection of several signaling molecules contributing to IRF3 activation including RIG-I, RIG-I-CARD (a constitutively active form of RIG-I), TRIFΔRIP, and cGAS/STING. The ability of UBCv1 to inhibit these pathways was assessed by its co-transfection alongside these inducers (Figure 2). In these experiments VACV protein C6, a well-known inhibitor of IRF3 signaling [32], was used as positive control. Expression of these signaling molecules resulted in clear induction of ISG56 reporter activity, and this was not affected by expression of UBCv1 or its mutant form (Figure 2A–C). We then analysed the effect of UBCv1 overexpression on an IFN-β promoter reporter. Upon stimulation with the inducers RIG-I, TRIFΔRIP, cGAS/STING or RIGI-CARD, the IFN-β-Luc reporter induction was significant, but this was not altered in cells overexpressing 3XFlag-UBCv1 or the mutant form with respect to EV (Figure 2D–F). Finally, to determine the role of UBCv1 on the JAK/STAT pathway, we used an ISRE-dependent luciferase reporter. Cells were co-transfected with the ISRE reporter and UBCv1, UBCv1mut or C6 as control and stimulated with IFNα for 8 h. Expression of C6 reduced ISRE activation as previously reported [35], but expression of UBCv1 had no significant inhibitory effect (Figure 2G).

### 3.3. UBCv1 Inhibits AP-1 Reporter Activity

**Figure 3:**
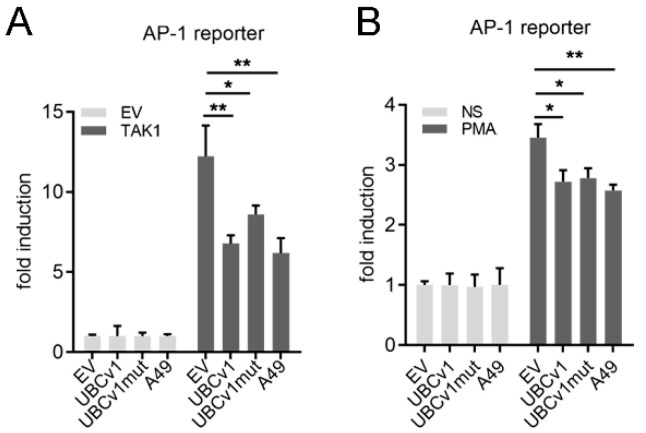
UBCv1 inhibited AP-1 transcription factor activation. HEK293T cells were transfected with pAP-1-Luc (250 ng/well), p-TK-Renilla (10 ng/well) and either 25 ng/well of UBCv1, UBCv1mut, A49 expression plasmids or empty vector (EV). (**A**) TAK1 (40 ng) inducer was also co-transfected. (**B**) 24 h after transfection, cells were treated with 10 ng/mL PMA for a further 8 h. Firefly luciferase activity was corrected with Renilla luciferase activity and normalized to non-stimulated (NS) empty vector (EV). Data are presented as mean ± SD and are representative of at least three experiments. Statistical analyses were performed using one-way ANOVA test (* *p* < 0.05; ** *p* < 0.01).

Transcriptional activity of activating protein-1 (AP-1) is regulated by cellular stress including viral infection [12], and AP-1/MAPK signaling contributes to type I IFN activation [36,37]. We therefore studied the effect of overexpressing UBCv1 on AP-1 transcription factor activation. First, cells were co-transfected with TAK1 inducers, UBCv1 constructs and a reporter expressing luciferase under the control of the AP-1 promoter (Figure 3). The signaling pathway was induced by expression of TAK1, which acts as a transactivating factor for both NF-κB and AP-1 pathways, and VACV protein A49 was used as a positive control [37]. As shown in Figure 3A, UBCv1 expression reduced significantly AP-1 induction compared to EV, as occurred in cells expressing VACV A49. Moreover, it was confirmed as stimulating the cells with PMA, which was reduced by co-expression of UBCv1 or UBCv1mut (Figure 3B). Together, these results indicated that both NF-κB and AP-1 but not IRF3 pathways were affected by UBCv1 overexpression in a manner that is independent of its ubiquitylation activity.

### 3.4. UBCv1 Inhibitory Activity Maps Upstream of the IKK Complex

**Figure 4:**
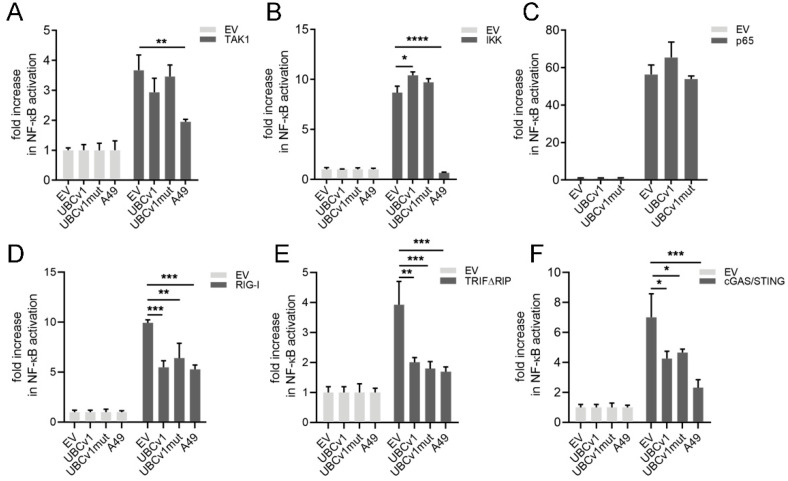
UBCv1 inhibitory activity mapped upstream of the IKK complex. (**A**) HEK293T cells were transfected with pNF-ĸB-Luc, p-TK-Renilla and UBCv1, UBCv1mut, positive control A49 expression plasmids or empty vector control (EV). Cells were stimulated by co-transfecting (**A**) TAK1 inducer (40 ng) (**B**) IKKβ signaling factor (50 ng) or (**C**) p65 (2 ng). Expression plasmids (**D**) RIG-I (40 ng), (**E**) TRIFΔRIP (40 ng) or (**F**) cGAS (20 ng) with STING (20 ng) were included as signaling factors in the initial transfection as indicated. Firefly luciferase activity was measured 24 h post- transfection and corrected with Renilla luciferase activity normalized to EV. Statistical analyses were performed using one-way ANOVA test (* *p* < 0.05; ** *p* < 0.01; *** *p* < 0.001; **** *p* < 0.0001).

The previous data indicated that UBCv1 inhibition could be exerted at the level of TAK1 and IKK complex. We therefore used TAK1 and the IKK catalytic subunit IKKβ as well as NF-ĸB transcription factor subunit p65 to activate an NF-ĸB-driven luciferase reporter and determine UBCv1 inhibitory potential. Ectopic expression of TAK1 and IKKβ resulted in activation of reporter activity and whilst this was blocked by VACV A49, we could not observe inhibition by UBCv1 (Figure 4A,B). In agreement with this, UBCv1 also failed to suppress NF-ĸB driven by p65 over-expression (Figure 4C). To further map UBCv1 activity, we then expressed RIG-I and TRIF, which activate NF-ĸB signaling upstream of the IKK complex in addition to IRF3. Co-expression of UBCv1 inhibited NF-ĸB activation driven by either RIG-I or TRIF (Figure 4D,E). Collectively, these results indicated that UBCv1 modulates NF-κB activation downstream TRAF6 and TRAF2 but upstream IKK complex. Also, our data showed that mutagenesis in the UBCv1 cysteine 85 catalytic residue did not interfere with the ability of UBCv1 to inhibit NF-κB inflammatory signaling overall.

### 3.5. UBCv1 Transient Expression Blocks p65 Translocation

**Figure 5:**
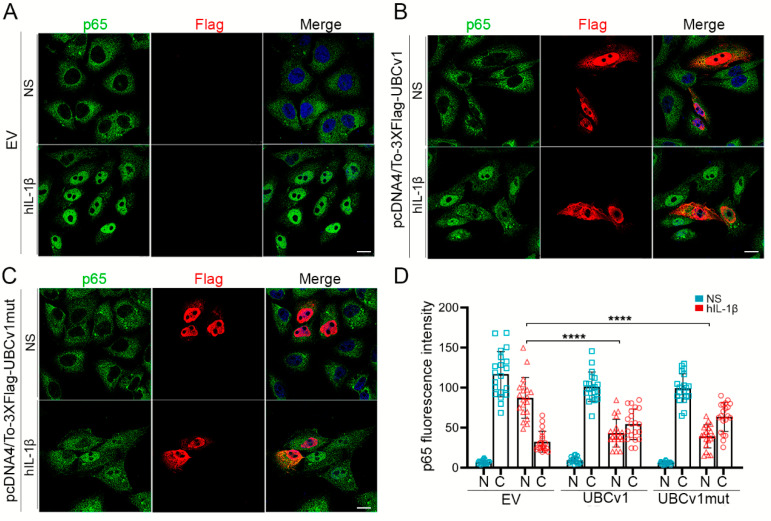
UBCv1 impaired p65 translocation into the nucleus. Representative images of HEK293T cells transfected with (**A**) pcDNA4/To (EV), (**B**) Flag-UBCv1 or (**C**) Flag-UBCv1mut and stimulated with IL-1β (50 ng/µL) for 30 min. Cells were then fixed, permeabilized and stained for Flag (green) and p65 (red). Non-stimulated (NS) transfected cells were used as controls. Protein localization was determined by confocal microscopy. Bar = 10 µm. (**D**) Quantification of p65 fluorescence intensity in nucleus (N) and cytoplasm (**C**) of transfected cells (EV, UBCv1 or UBCv1mut) in NS and hIL-1β conditions (*n* = 20). Statistical analyses were performed using one-way ANOVA test (**** *p* < 0.0001).

In resting cells, IκBα is found in a complex with NF-κB transcription factor subunits p65 and p50 in the cytoplasm, preventing their nuclear translocation and subsequent activation of NF-κB-dependent gene transcription [38]. As we found UBCv1 could be acting upstream or at the level of IKK, we next assessed p65 translocation into the nucleus in these cells. Vero cells were transfected for 24 h with the empty vector (EV), 3XFlag-UBCv1 or 3XFlag-UBCv1mut plasmids and then, stimulated for 30 min with IL-1β to induce the translocation of p65 from the cytoplasm to the nucleus. In non-stimulated cells (NS), p65 localized in the cytoplasm of cells overexpressing UBCv1 or not (Figure 5A–C, upper panels). However, after IL-1β treatment, p65 translocated into the nucleus in EV-transfected cells (Figure 5A), while it was clearly retained in the cytoplasm of cells overexpressing UBCv1 (red; Figure 5B lower panel). We observed a similar pattern when cells overexpressed UBCv1mut (Figure 5C, lower panel). To validate the UBCv1 ability to inhibit p65 translocation, we quantified the fluorescence signal intensity obtained in each condition (Figure 5D). This determination confirmed that UBCv1 impaired p65 translocation and its mutation at the Cys 85 did not interfere its capacity of NF-κB inhibition. Together, these results indicated that UBCv1 has the ability to inhibit p65 translocation downstream of IL-1β stimulation.

### 3.6. UBCv1 Overexpression Reduces Levels of NF-κB Dependent Genes

**Figure 6:**
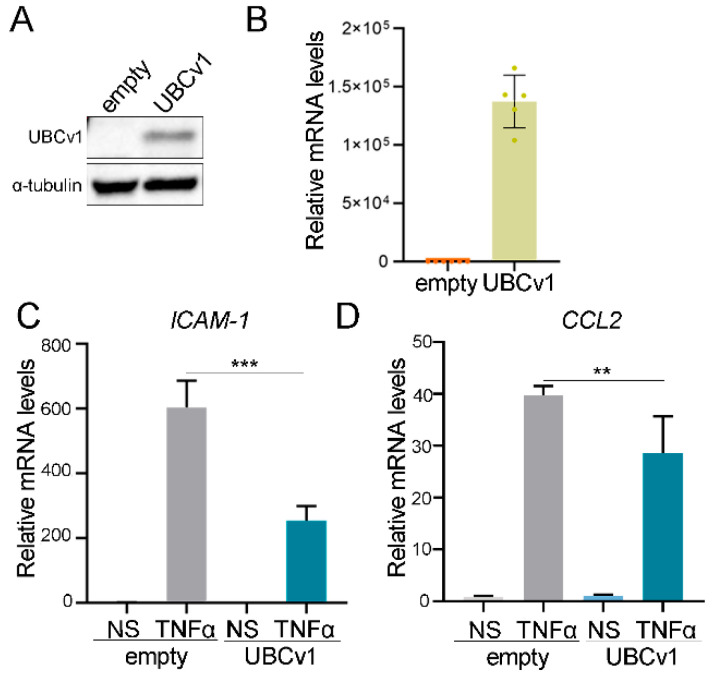
UBCv1 downregulated the expression of NF-ĸB dependent genes. A549- empty and A549-UBCv1 stable cell lines were assessed for expression by (**A**) western blot (also Figure S1) and (**B**) quantitative PCR using UBCv1 antibody and UBCv1 primers, respectively. (**C**,**D**) Stable cell lines were stimulated for 6 h with 25 ng of TNF-α, and total RNA was extracted and analysed by qPCR using *ICAM-1* or *CCL*-two specific primers. *GAPDH* levels were used as control. Data are presented as fold increase over non-stimulated cells (NS) for each cell line and representative of three experiments performed in triplicate. Statistical analyses were performed using one-way ANOVA test. (** *p* < 0.01; *** *p* < 0.001).

Finally, to confirm these data, we generated an A549 cell line stably expressing UBCv1 as described in the Materials and Methods section. First, we validated these cells lines (control and UBCv1) by western blot and quantitative PCR (qPCR). Expression of UBCv1 was readily detectable (Figure 6A,B). Then, we studied the expression of *ICAM-1* and *CCL-2*, genes with NF-ĸB dependent promoters. A549-empty and A549-UBCv1 cells were stimulated with TNF-α for 6 h and *ICAM-1* and *CCL-2* were measured by qPCR. The expression levels of these genes were reduced in UBCv1-expressing cells compared to control cells (Figure 6C,D), confirming the ability of UBCv1 to block NF-κB transcriptional activity.

## 4. Discussion

More than half of the 150 genes encoded by ASFV are not required for virus replication, but have an important role in evading host defences [39]. In this work, our interest was directed towards the viral protein UBCv1 due to the importance of ubiquitin enzymes in the regulation of the immune response [40]. Innate immune activation in response to invading viral pathogens initiates through the engagement of PRR(s) and the subsequent activation of multiple different intracellular signaling pathways. Critical amongst these is the activation of NF-κB, AP-1 and IRF-3/7, which mediate the induction of type I IFN. Here, we provide evidence demonstrating that UBCv1 is a previously unrecognized inhibitor of NF-κB activation. UBCv1 suppressed NF-ĸB-dependent transcription in response to TNFα, IL-1β and several signaling molecules, and prevented p65 nuclear translocation. Experiments aiming to map this inhibitory activity indicated that UBCv1 acts upstream of the IKK complex. This would concur with the NF-κB inhibition detected in the presence of cytosolic acid nucleic sensors, such as cGAS/STING, TRIF and RIG-I, given that their signaling pathways induce NF-κB activation through TAK1 and IKKβ.

ASFV has adapted several genes and proteins to control inflammatory responses and specifically NF-κB. ASFV protein A238L acts as a homologue of IκB and inhibits NF-κB as well as NFAT transcription factor activation. However, it apparently did not affect AP-1 transcription factor [17,18]. Our results indicate that UBCv1 (I215L) is a new NF-ĸB modulator that cooperates with A238L. The presence of multiple viral proteins targeting the same innate immune pathway providing functional redundancy is not uncommon, and has been extensively reported for another large DNA virus such as VACV [39,41,42,43]. For instance, VACV protein A49 (used here as a control for NF-κB activation assays) acts at the level of IκBα degradation, blocking this by virtue of an N-terminal extension that mimics the IκBα phosphodegron recognised by the E3 ubiquitin ligase β-TrCP [30,34,44,45]. Upstream of A49, several other viral proteins have the capacity to suppress NF-κB activation, acting at the level of TLR adaptors, TRAFs and IKK (reviewed in [41,42]). The value of encoding for these viral upstream inhibitory molecules is thought to confer a stronger immunomodulatory potential not only due to the cumulative action of these proteins on one pathway, but also on other crosstalking pathways. Therefore, it appears that both ASFV and VACV have evolved strategies to redundantly target NF-κB signaling. Given the coding capacity of ASFV and the importance of NF-κB in the antiviral response, it is likely that other viral immunosuppressive proteins remain to be identified.

ASFV infection of host cells also induces the transcription of multiple cellular genes, including interferons (IFNs). Interferon regulatory factor 3 (IRF-3) is directly activated upon virus infection and functions as a key activator of interferon (IFN) genes [13]. Viral protein I329L inhibits IFN-β induction and IRF3 activation at the level of TRIF [23]. In contrast with the inhibitory capacity of genes such as VACV C6 [32], UBCv1 was not able to interfere with IFN-β promoter activation in the conditions tested. A similar absence of inhibitory activity was observed for ISRE signaling. Further work is required to confirm the inability of UBCv1 to modulate these signaling pathways.

The results presented here indicate that UBCv1 mutated in the Cys85 catalytic site had no effect in the NF-κB inhibitory role of UBCv1. UBCv1 acts as an E2 Ub enzyme in vitro, and it needs Cys85 for the transthioesterification reaction and it is capable to bind several types of ubiquitin chains [27,28]. These observations suggest that UBCv1 holds at least two independent functions, ubiquitylation and NF-κB suppression. It is possible that UBCv1 binds an E3 ubiquitin ligase complex important for NF-κB activation blocking its action. Indeed, this is the case of VACV A49 and the β-TrCP ubiquitin ligase. Our data indicate that UBCv1 does not act at the level of β-TrCP, but upstream of the IKK complex, suggesting that other E3 ubiquitin ligases acting at that level could be the target of UBCv1. Recent unbiased analysis of Cullin-5 E3 ubiquitin ligases has identified novel positive regulators of NF-κB signaling that were previously unnoticed [29]. This indicates that many different host ubiquitin ligases modulate inflammatory responses and studying viral targeting may reveal its identity. It is also possible that UBCv1 association with a yet unknown E3 ligase redirects its ubiquitylation activity towards substrates that are important for other cellular pathways. Such mechanism would allow to manipulate not only NF-κB activity, but other pathways in a manner that would be beneficial for the virus. The identity of these other pathways remains unknown and will be the focus of future work.

The study of viral immunomodulation has the potential to yield fundamental insights into the regulation of inflammation and how cells respond to viral infection. The current COVID-19 pandemic has spotlighted that aberrant activation of NF-ĸB and inflammatory signaling could result in severe disease, with high morbidity and mortality. A better understanding of how these pathways are activated and regulated is therefore needed for the development of novel therapeutics. ASFV is a swine virus for which no vaccine has been approved yet. The development of attenuated vaccines in which specific immunomodulators are removed is a promising approach [15,16]. Work with other large DNA viruses has demonstrated that the removal of NF-κB inhibitors enhances the development of effector and memory T cell responses, and provides safer and yet more immunogenic vaccine vectors [46,47,48], although multiple deletions have been shown to reduce immunogenicity [49]. These observations indicate that detailed molecular understanding of viral immunomodulators is required to inform the design of rationally attenuated vaccines.

Collectively, we have described here the ability of ASFV ubiquitin-conjugating enzyme UBCv1 to dampen inflammatory signaling by inhibiting NF-κB and AP-1 signaling pathways. Our results reveal a novel function for this important viral protein and provide a new avenue of research to understand ASFV immune evasion of host antiviral responses.

## Data Availability

Not applicable.

## Supplementary Materials

The following are available online at https://www.mdpi.com/article/10.3390/v13061160/s1, Figure S1: Uncropped full length pictures of Western blotting membranes from Figure 6.
