# Supplementary figures and images for: African Swine Fever Virus Ubiquitin-Conjugating Enzyme Is an Immunomodulator Targeting NF-κB Activation
Source: Viruses. 2021 Jun 17;13(6):1160. doi: 10.3390/v13061160 (PMC8233900; doi:10.3390/v13061160)

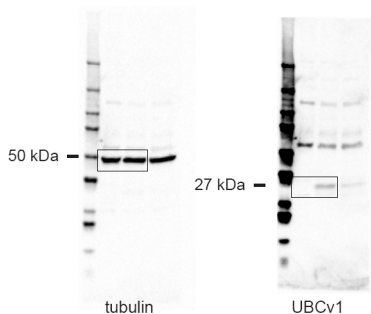

Figure S1. Uncropped full-length pictures of western blotting membranes from Figure 6.

Supplement: Supplementary file 1 [file viruses-13-01160-s001.zip › viruses-1241565-supplementary.pdf]
